# Non-Iterative Reconstruction and Selection Network-Assisted Channel Estimation for mmWave MIMO Communications

**DOI:** 10.3390/s25134172

**Published:** 2025-07-04

**Authors:** Jing Yang, Yabo Guo, Xinying Guo, Pengpeng Wang

**Affiliations:** 1 Key Laboratory of Grain Information Processing and Control (Henan University of Technology), Ministry of Education, Zhengzhou 450001, China; 2 Henan Key Laboratory of Grain Photoelectric Detection and Control, Henan University of Technology, Zhengzhou 450001, China; 3 Henan Engineering Research Center of Grain Condition Intelligent Detection and Application, Henan University of Technology, Zhengzhou 450001, China; ybguo@haut.edu.cn (Y.G.); guoxinying@haut.edu.cn (X.G.); wangpp5577@163.com (P.W.)

**Keywords:** beamspace, mmWave MIMO, channel estimation, non-iterative reconstruction network (NIRNet), selection matrix

## Abstract

Millimeter-wave (mmWave) MIMO systems have emerged as a key enabling technology for next-generation wireless networks, addressing the growing demand for ultra-high data rates through the utilization of wide bandwidths and large-scale antenna configurations. Beyond communication capabilities, these systems offer inherent advantages for integrated sensing applications, particularly in scenarios requiring precise object detection and localization. The sparse mmWave channel in the beamspace domain allows fewer radio-frequency (RF) chains by selecting dominant beams, boosting both communication efficiency and sensing resolution. However, existing channel estimation methods, such as learned approximate message passing (LAMP) networks, rely on computationally intensive iterations. This becomes particularly problematic in large-scale system deployments, where estimation inaccuracies can severely degrade sensing performance. To address these limitations, we propose a low-complexity channel estimator using a non-iterative reconstruction network (NIRNet) with a learning-based selection matrix (LSM). NIRNet employs a convolutional layer for efficient, non-iterative beamspace channel reconstruction, significantly reducing computational overhead compared to LAMP-based methods, which is vital for real-time sensing. The LSM generates a signal-aware Gaussian measurement matrix, outperforming traditional Bernoulli matrices, while a denoising network enhances accuracy under low SNR conditions, improving sensing resolution. Simulations show the NIRNet-based algorithm achieves a superior normalized mean squared error (NMSE) and an achievable sum rate (ASR) with lower complexity and reduced training overhead.

## 1. Introduction

As a core technology of 5G, millimeter-wave (mmWave) multiple-input multiple-output (MIMO) can offer ultra-high data rates by combining wide bandwidth and large-scale antenna arrays while also enabling integrated sensing capabilities for applications like object detection and localization through integrated sensing and communications (ISAC). This enables high spectral efficiency and user capacity but also introduces challenges such as severe path loss, complex channel estimation, and the need for accurate real-time processing to support both communication and sensing tasks [1,2,3,4].

To fully exploit the potential of mmWave communications, the accurate acquisition of channel state information (CSI) is essential. However, in mmWave massive MIMO systems, the high dimensionality of the channel poses significant challenges for efficient CSI estimation. A straightforward solution employing a dedicated radio-frequency (RF) chain for each antenna is often impractical due to excessive hardware cost and power consumption [5]. To address this issue, a lens antenna array was proposed in ref. [6] to transform the spatial channel into a beamspace representation by focusing signals from different directions onto distinct antennas. Owing to the limited number of effective propagation paths in mmWave frequencies, the beamspace channel exhibits inherent sparsity. Consequently, by selecting only a few dominant beams, the number of required RF chains can be substantially reduced, thereby facilitating the development of low-complexity channel estimation methods.

Despite advancements, channel estimation in beamspace mmWave massive MIMO systems remains a challenging problem, particularly for large-scale lens antenna arrays with a limited number of RF chains. The work in ref. [7] proposed a nonparametric channel estimation algorithm for massive massive multiuser MIMO mmWave systems, which can achieve MSE-optimal performance in the large-antenna limit. A novel model-driven unsupervised deep learning (DL) network for wideband mmWave beamspace channel estimation was proposed in ref. [8], which inherits the superiority of iterative signal recovery algorithms and the advanced DL-based denoiser and thus presents excellent performance. Levering the advantage of deep learning networks, the work in refs. [9,10] respectively proposed the approximate message passing-aided algorithm to solve the beamspace channel estimation problem in mmWave massive MIMO systems, which can achieve better performance with much lower training complexity compared with the existing methods. Based on tensor-based ESPRIT-type algorithms in DFT beamspace, the work in ref. [11] developed a gridless channel estimation algorithm for a hybrid mmWave MIMO-OFDM system with a frequency selective channel, where the proposed algorithm can provide accurate channel estimates with only a few training resources. Exploiting the unique power distribution (PD) of the beamspace channel (BC) that differs from the general sparse signals, ref. [12] proposed a PD-based estimation scheme for the sparse BC. Ref. [13] presented an SBL approach for channel estimation in mmWave hybrid MIMO OFDM systems that exploits the sparsity of the beamspace mmWave channel for each individual subcarrier. In ref. [14], by considering the potential multiuser interferences, an IA beam selection consisting of two stages to achieve the near-optimal sum-rate performance with low complexity was proposed. Simulation results verify that the proposed interference-aware beam selection can achieve the sum-rate performance close to the fully digital system with much higher energy efficiency. By exploiting the inherent sparsity of the beamspace channel matrix, a support detection (SD) algorithm was proposed in ref. [15], which applies compressed sensing (CS) techniques and achieves better normalized mean squared error (NMSE) performance than the classical orthogonal matching pursuit (OMP) algorithm. However, the SD algorithm mainly leverages sparsity and fails to utilize the energy-focusing property of the lens antenna array. To overcome this limitation, an alternative method based on approximate message passing (AMP), called the sparse non-informative parameter estimator-based cosparse analysis AMP for imaging (SCAMPI), was proposed in ref. [16]. SCAMPI outperforms both OMP and SD in terms of NMSE. Nevertheless, its estimation accuracy degrades significantly in low signal-to-noise ratio (SNR) scenarios with the NMSE reaching a suboptimal level of 3.5 dB at an SNR of 5 dB. To further enhance the NMSE performance at low SNRs, a learned denoising-based AMP algorithm (LDAMP) is introduced for beamspace mmWave massive MIMO systems in ref. [17]. This approach integrates a denoising convolutional neural network (DnCNN) into the AMP framework, achieving superior NMSE performance compared to the SCAMPI algorithm even with a reduced number of RF chains. Building on these developments, a fully convolutional denoising AMP algorithm (FCDAMP) was proposed in ref. [18], which integrates a fully convolutional denoising network with a learned AMP (LAMP) framework. FCDAMP achieves further improvements in NMSE performance compared to LDAMP. Furthermore, an AMP-based network with deep residual learning, termed LampResNet, was introduced in ref. [19]. By combining a LAMP architecture with residual learning techniques, LampResNet not only surpasses LDAMP in terms of NMSE but also achieves reduced computational complexity. Despite these advancements, the LAMP networks employed in both FCDAMP and LampResNet algorithms rely on high-complexity iterative operations. Furthermore, in beamspace mmWave massive MIMO systems, the effectiveness of the selection matrix in preserving the structural characteristics of the channel matrix during the compression of the received signal matrix significantly impacts the accuracy of channel reconstruction. A notable limitation of existing selection matrices is their reliance on randomly generated Bernoulli matrices, which are independent of the signal characteristics. This approach often results in suboptimal channel reconstruction accuracy.

To address the limitations of existing channel estimation methods in beamspace mmWave massive MIMO systems, we propose a novel low-complexity channel estimator that leverages the NIRNet and a learning-based selection matrix (LSM). This approach fully exploits the structural characteristics of the channel matrix to achieve superior performance. By integrating the LSM, which is optimized to preserve critical channel features, and the NIRNet, which employs non-iterative convolutional techniques for efficient reconstruction, the proposed method significantly enhances reconstruction accuracy while maintaining low computational complexity. Simulation results demonstrate that the NIRNet-based channel estimation algorithm outperforms the benchmarks in terms of NMSE and ASR. Additionally, the proposed method exhibits lower computational complexity, making it a highly efficient solution for beamspace mmWave massive MIMO systems. The contributions of this paper may be summarized as follows:

- Unlike conventional Bernoulli selection matrices, the LSM is trained on prior data with its elements adhering to a Gaussian distribution whose statistical properties are progressively optimized during training. Consequently, the measurement matrix produced by the LSM preserves a greater number of structural features of the channel matrix, significantly enhancing reconstruction quality.
- In contrast to LAMP-based algorithms, which rely on computationally intensive iterative operations for reconstruction, the proposed NIRNet algorithm employs a convolutional layer to perform initial channel matrix reconstruction in a non-iterative manner. The reconstruction accuracy is further improved by incorporating a denoising network, which refines the channel estimate, thereby achieving superior performance with reduced computational overhead.

The remainder of this paper is organized as follows: Section 2 introduces the beamspace mmWave MIMO system model and channel characteristics, which is followed by a formal formulation of the channel estimation problem. Section 3 presents the novel NIRNet-based channel estimator in detail, elaborating on the architecture, operational principles, and theoretical foundations of the proposed algorithm. Comprehensive simulation results are presented in Section 4, where we evaluate the performance of our approach against existing methods using various metrics and analyze the computational complexity advantages of our solution. Finally, Section 5 summarizes our findings and discusses the implications of this work.

*Notation*: Throughout the paper, we use the following notations. Boldface lowercase and uppercase letters denote vectors and matrices, respectively. A Gaussian distribution of *x* with mean $\hat{x}$ and variance $\nu_{x}$ is represented by $\mathrm{CN}(x;\hat{x},\nu_{x})$. The notation ||A|| is the Frobenius norm of A. We use 1, 0 and I to denote an all-one matrix, an all-zero matrix and an identity matrix with a proper size, respectively.

## 2. System Model and Problem Formulation

**Figure 1:**
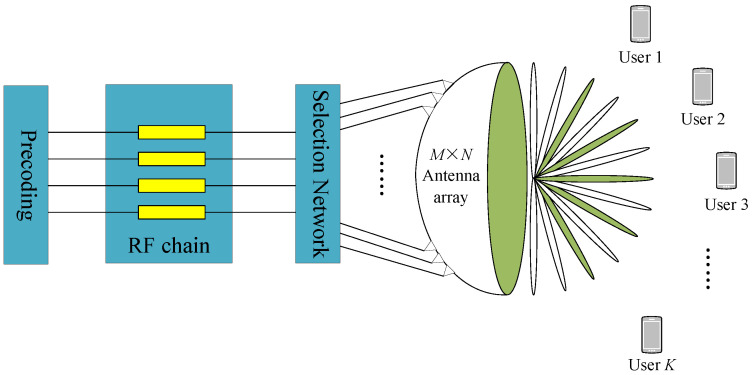
The beamspace mmWave massive MIMO system architecture.

We consider a time division duplexing (TDD) beamspace mmWave MIMO system. As depicted in Figure 1, the base station (BS) is equipped with a single lens antenna array comprising an M×N antenna, which is interfaced with $N_{\mathrm{RF}}$ radio-frequency (RF) chains to concurrently serve *K* single-antenna users. To mitigate the hardware costs and power consumption associated with RF chains, we constrain the number of RF chains such that $N_{\mathrm{RF}}\ll MN$ while ensuring $N_{\mathrm{RF}}\geq K$ to maintain the spatial multiplexing gain for *K* users [15]. For optimal efficiency, we focus on channel estimation utilizing the minimum number of RF chains, specifically setting $N_{\mathrm{RF}}=K$, thereby balancing cost, power efficiency, and system performance.

We adopt the Saleh–Valenzuela channel model for the beamspace mmWave massive MIMO system, as described in ref. [15]. The beamspace channel matrix for the *k*-th user (k∈1,2,…,K), denoted as $H_{k}\in C^{M\times N}$, is expressed as

(1) $$H_{k}=\sqrt{\frac{MN}{L+1}}\sum_{i=0}^{L}\alpha^{\left(i\right)}A\left(\varphi^{\left(i\right)},\theta^{\left(i\right)}\right),$$

where L+1 represents the total number of propagation paths, $\alpha^{\left(i\right)}$ denotes the complex gain of the *i*-th path, and $\varphi^{\left(i\right)}$ and $\theta^{\left(i\right)}$ indicate the azimuth and elevation angles of the corresponding plane wave, respectively. The antenna array response matrix $A(\varphi^{\left(i\right)},\theta^{\left(i\right)})$ encapsulates the spatial characteristics of the lens antenna array. The element in the *m*-th row and *n*-th column of A is given by ref. [20]

(2) $$A_{m,n}\left(\varphi^{\left(i\right)},\theta^{\left(i\right)}\right)=\sqrt{D}\sin c\left(m-D_{M}\sin\left(\varphi^{\left(i\right)}\right)\right)\times \sin c\left(n-D_{N}\sin\left(\theta^{\left(i\right)}\right)\right),$$

where *D* denotes the aperture length of the antenna, and $D_{M}$ and $D_{N}$ represent the antenna position indices along the horizontal and vertical axes of the lens antenna array, respectively. To facilitate processing, $H_{k}$ can be vectorized to a vector $h_{k}\in C^{MN\times 1}$, and the overall channel matrix H can be defined as $H≜\left[h_{k},\ldots ,h_{K}\right]\in C^{MN\times K}$.

In the uplink of a TDD system, all *K* users transmit pilot sequences to the BS within the channel coherence time. We assume the beamspace channel remains constant during this period [21]. Each user sends *K* pilot symbols to the BS, and the pilot matrix $S\in C^{K\times K}$ comprises *K* mutually orthogonal pilot sequences: one per user [21,22]. To standardize the uplink pilot power, the pilot matrix satisfies $SS^{H}=I_{K}$, where $I_{K}$ is the K×K identity matrix. Thus, the received signal matrix at the BS, $Y\in C^{MN\times K}$, can be expressed as the received signal matrix at the BS, which is is given by

(3) Y=HS+N,

where $H\in C^{MN\times K}$ represents the beamspace channel matrix for *K* users, and $N\in C^{MN\times K}$ denotes the additive white Gaussian noise (AWGN), with elements independently drawn from a complex normal distribution $\mathrm{CN}(0,\sigma^{2})$, where $\sigma^{2}$ is the noise variance. During the pilot transmission phase, BS employs a selection matrix $W\in C^{K\times MN}$ to compress the received signal Y, which can be denoted as $R\in C^{K\times K}$, i.e.,

(4) R=WY=WHS+WN.

By multiplying $S^{H}$ on the right side of (3), the measurement matrix $Z\in C^{K\times K}$ is obtained, which is expressed as

(5) $$Z=RS^{H}=WH+\tilde{W},$$

where $\tilde{W}≜WNS^{H}$ is the effective noise matrix.

We focus on estimating the channel matrix H from the observed matrix Z. Given the large-scale nature of the antenna array, particularly in systems with dimensions in the order of M×N, the computational complexity of channel estimation becomes a critical challenge for practical implementation. Traditional algorithms for channel estimation, such as least squares (LS) and LAMP, typically suffer from high computational complexity due to their iterative nature, rendering them computationally prohibitive for massive MIMO systems operating at mmWave frequencies. To overcome this challenge, we reformulate the beamspace channel estimation problem as a sparse signal recovery task. We introduce a novel, non-iterative reconstruction network that incorporates a learning-based selection matrix, which significantly reduces the computational overhead while maintaining high estimation accuracy.

## 3. NIRNet-Based Channel Estimation

**Figure 2:**
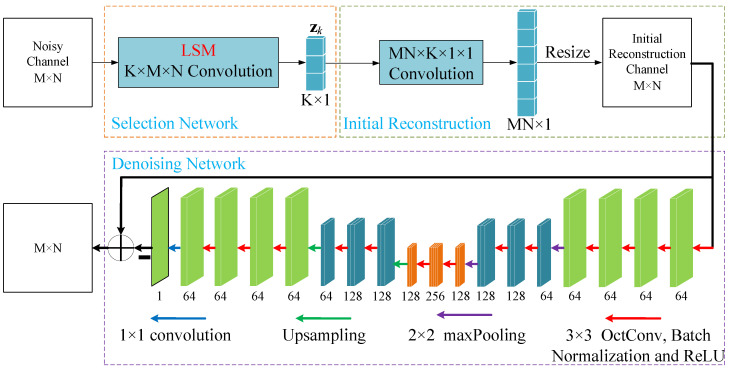
The NIRNet-based channel estimation structure.

In this section, we introduce the NIRNet algorithm as an advanced solution for channel matrix estimation in massive MIMO systems. This approach reconstructs the channel matrix non-iteratively, leveraging prior information from transmitted data to enhance estimation efficiency and accuracy. The NIRNet framework comprises three integral components, as illustrated in Figure 2. First, the selection network is trained to generate an LSM, optimizing the sparse representation of the channel by identifying and prioritizing the most informative beamspace components, thus enhancing the efficiency of subsequent estimation tasks. Next, the reconstruction network produces an initial channel matrix estimate through a single forward pass, utilizing the LSM to capture key channel characteristics while significantly lowering computational complexity compared to conventional iterative approaches. Finally, the denoising network refines this initial estimate by suppressing noise, artifacts, and residual errors, thereby improving reconstruction quality and ensuring robust performance in challenging mmWave environments. In the following sections, we elaborate on the operation of the NIRNet-based channel estimation algorithm.

To reconstruct the beamspace channel matrix H from the measurement matrix Z, as outlined in Section 2, the selection matrix W is designed to minimize the mutual coherence, which is defined as

(6) $$\mu ≜\max_{i\neq j}|w_{i}^{H}w_{j}|,$$

where $w_{i}$ represents the *i*-th column of W. By reducing μ to the smallest possible value, the selection matrix enhances the sparsity of the channel representation, thereby improving the accuracy and computational efficiency of the recovery process in massive MIMO systems. Prior studies, such as [23], have demonstrated that Gaussian and Bernoulli random matrices exhibit low mutual coherence, making them suitable candidates for W. However, these matrices are signal-independent and thus fail to exploit prior information about the channel, potentially limiting their performance in scenarios where such information is available. To address this limitation, we propose a learning-based approach to construct W, which adapts to the signal characteristics and incorporates prior knowledge, further optimizing the channel estimation process.

To effectively exploit the prior information embedded in the transmitted data, the NIRNet algorithm designs the selection matrix W as a convolutional layer within a dedicated selection network. The convolutional kernel size is tailored to match the dimensions of the beamspace channel matrix for the *k*-th user, ensuring compatibility with the channel’s sparse structure. The number of feature maps in this layer is set to *K*, corresponding to the number of users or signal streams. Use $z_{k}$ to denote the *k*-th volume of the matrix Z, and it can be formulated as

(7) $$z_{k}=\mathrm{conv}\left(F,H_{k}\right)=F*H_{k},$$

where F denotes the convolutional kernel of the layer. To promote robustness and sparsity, each weight of F is independently drawn from a complex Gaussian distribution with zero mean and variance 1/K, which is expressed as shown below:

(8) $$F_{i,j}∼\mathrm{CN}\left(0,\frac{1}{K}\right).$$

**Figure 3:**
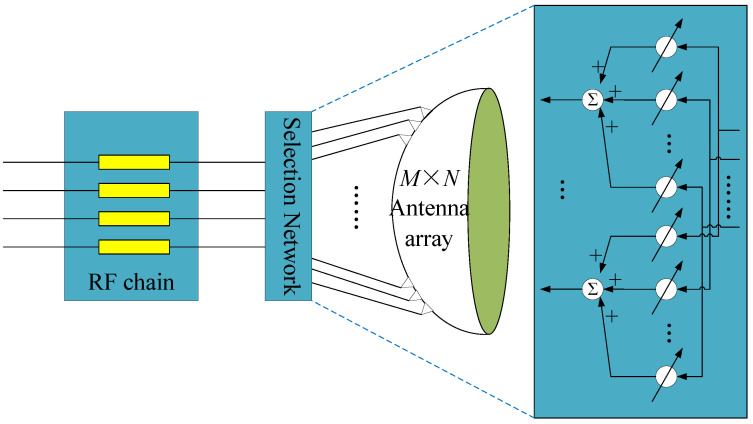
The proposed combination selection network for a beamspace mmWave massive MIMO.

Through an optimized training phase, the selection network generates an LSM, which is specifically adapted to the beamspace characteristics of the mmWave massive MIMO system, as illustrated in Figure 3. The proposed LSM implementation employs a hardware-efficient scheme, utilizing multipliers and adders to compute the product of the selection matrix W and the received signal matrix during beamspace channel estimation. This approach not only leverages prior information to enhance estimation accuracy but also minimizes computational complexity, making it well suited for practical deployment in large-scale mmWave systems.

Unlike conventional Gaussian and Bernoulli random matrices, which are signal-independent, the LSM is derived through a data-driven training process. During training, the elements of the LSM are iteratively optimized to minimize the loss function, enabling the LSM to capture and preserve the structural features of the beamspace channel matrix $H_{k}$ within the received signal $z_{k}$. This adaptive approach ensures that $z_{k}$ retains critical channel characteristics, enhancing the accuracy of subsequent channel estimation in mmWave massive MIMO systems. In the reconstruction network, the channel matrix is estimated non-iteratively to achieve computational efficiency. Specifically, a single convolutional layer with a 1 × 1 kernel is employed, where the number of feature maps corresponds to the total number of base station antennas, i.e., MN. The reconstruction process is formulated as a convolution operation

(9) $${\hat{h}}_{\mathrm{int}}=\mathrm{conv}\left(F_{\mathrm{int}},z_{k}\right)=F_{\mathrm{int}}*z_{k},$$

where ${\hat{h}}_{\mathrm{int}}$ denotes the initial reconstructed channel vector of dimension 1×MN, and $F_{\mathrm{int}}$ represents the convolutional kernel. The vector ${\hat{h}}_{\mathrm{int}}$ is subsequently reshaped into an M×N matrix, denoted as ${\hat{H}}_{\mathrm{int}}$, to represent the initial channel matrix estimate. However, relying solely on the reconstruction network is insufficient, as it does not address the impact of channel noise and interference prevalent in mmWave environments. To mitigate these effects, the NIRNet algorithm incorporates a dedicated denoising network as an integral component. This network refines the initial estimate ${\hat{H}}_{\mathrm{int}}$ by suppressing noise and artifacts, thereby improving the overall quality and reliability of the channel reconstruction. The combination of these tailored components ensures that NIRNet achieves both high accuracy and computational efficiency, making it well suited for practical deployment in large-scale mmWave MIMO systems.

The denoising network in the NIRNet algorithm adopts a modified U-Net architecture [24], which is designed to refine the initial reconstruction channel matrix ${\hat{H}}_{\mathrm{int}}$ and produce a high-fidelity estimated channel matrix ${\hat{H}}_{k}$. As depicted in Figure 2, the network comprises 14 convolutional layers, two max-pooling layers, and two transposed convolutional layers, which were strategically organized to balance computational efficiency and performance. Unlike the conventional U-Net, the first 13 convolutional layers utilize octave convolution [25], which reduces computational complexity by processing features at multiple frequency resolutions. The network operates through a contracting path, which extracts hierarchical features, and an expanding path, which restores the feature map to its original dimensions. The contracting path consists of repeated blocks, each containing two octave convolution layers followed by a max-pooling layer, which are described by the operations $G_{\mathrm{Oct}}$ and $G_{\mathrm{MP}}$, respectively

(10) $$G_{\mathrm{Oct}}\left(\hat{x}\right)=\mathrm{ReLU}\left(\mathrm{OctConv}(\hat{x},3)\right),$$

(11) $$G_{\mathrm{MP}}\left(\hat{x}\right)=\mathrm{MaxPool}\left(\hat{x},2\right),$$

where $\hat{x}$ represents the input feature maps, OctConv(·,3) denotes a 3 × 3 octave convolution, ReLU(·) is the activation function, and MaxPool(·,2) indicates a 2 × 2 max-pooling operation. The expanding path mirrors this structure, incorporating two octave convolution layers (as in Equation (9)) and one transposed convolution layer, which is defined as

(12) $$G_{\mathrm{TC}}\left(\hat{x}\right)=\mathrm{ReLU}\left(\mathrm{TransConv}(\hat{x},2)\right),$$

where TransConv(·,2) represents a 2 × 2 transposed convolution. In the contracting path, the number of feature channels doubles to capture richer features, while in the expanding path, it is halved to reconstruct the output. Batch normalization is applied after each octave convolution layer to accelerate training convergence and enhance model stability. The denoising network subtracts the estimated noise from ${\hat{H}}_{\mathrm{int}}$, yielding the refined channel matrix ${\hat{H}}_{k}$.

## 4. Simulation Results

In this section, we evaluate the performance of the NIRNet algorithm for the *k*-th user. The simulation parameters used in the experiments are summarized in Table 1. The number of antennas at the BS is set to 64, while each user is equipped with a single antenna. The number of propagation paths is set to 3. The azimuth and elevation angles, denoted by $\phi^{\left(i\right)}$ and $\theta^{\left(i\right)}$, are independently and uniformly distributed in the range $\left[-\frac{\pi }{2},\frac{\pi }{2}\right]$. The path gain $\alpha^{\left(i\right)}$ is modeled as a complex Gaussian random variable with zero mean and unit variance, i.e., $\alpha^{\left(i\right)}∼\mathrm{CN}\left(0,1\right)$. The training rate is set to 0.001. The dataset consists of 16,000 training samples and 8000 testing samples. The mini-batch size used during training is 128. To assess the algorithm’s performance, we utilize two distinct mean absolute error (MAE) loss functions, denoted as Loss1 and Loss2, which are defined as follows

(13) $$\mathrm{Loss}1=\frac{1}{MN}\sum_{i=1}^{M}\sum_{j=1}^{N}|{\left[H_{k}\right]}_{i,j}-{\left[{\hat{H}}_{k}\right]}_{i,j}|,$$

(14) $$\mathrm{Loss}2=\frac{1}{MN}\sum_{i=1}^{M}\sum_{j=1}^{N}|{\left[H_{k}\right]}_{i,j}-{\left[{\hat{H}}_{\mathrm{int}}\right]}_{i,j}|.$$

Loss1 is used to train the entire NIRNet model, guiding the optimization of the overall channel estimation framework. On the other hand, Loss2 is specifically applied during the training of the reconstruction network, focusing on refining the accuracy of the estimated channel matrix. The NIRNet algorithm is optimized using the Adam optimizer, which is a widely adopted gradient-based optimization technique known for its efficiency and adaptive learning rate capabilities. To evaluate the performance of the channel estimation, we utilize the Normalized Mean Square Error (NMSE) as the performance metric, which provides a quantitative measure of the accuracy of the estimated channel matrix. The NMSE is defined as

(15) $$\mathrm{NMSE}=E\left\{∥\hat{H}-H{∥}_{F}^{2}/∥\hat{H}{∥}_{F}^{2}\right\}.$$

**Figure 4:**
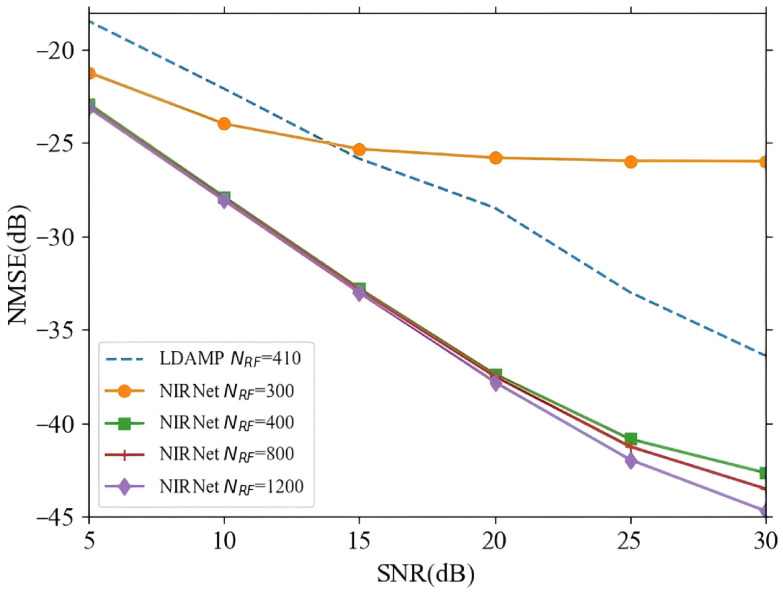
The NMSE performance of NIRNet for varying numbers of RF chains.

Figure 4 illustrates the NMSE performance of the proposed NIRNet and LDAMP algorithms versus SNR as the number of RF chains increases. The NIRNet algorithm demonstrates superior NMSE performance even with a lower number of RF chains under low SNR conditions. For instance, at an SNR range of 5–10 dB, NIRNet requires only 300 RF chains to achieve better NMSE performance than LDAMP [17], which utilizes 410 RF chains. It is also noteworthy that NIRNet with 400 RF chains significantly outperforms LDAMP with 410 RF chains. Additionally, the proposed algorithm exhibits convergence in terms of NMSE performance when the number of RF chains reaches 400. This implies that increasing the number of RF chains beyond 400 yields only marginal improvements in performance. Therefore, the performance of NIRNet with 400 RF chains is notably superior to that with 300 RF chains, and increasing the number of RF chains to 800 provides only slight improvements. This finding highlights that the NIRNet algorithm does not require an excessively large number of RF chains, thus offering a more efficient solution by reducing both hardware costs and power consumption.

**Figure 5:**
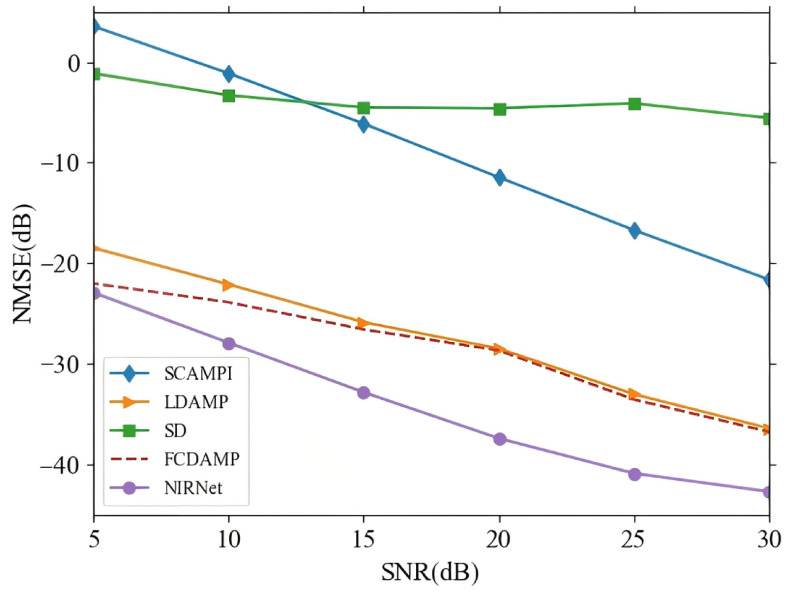
NMSE performance comparison between NIRNet and alternative algorithms.

Figure 5 presents a comparison of the NMSE performance versus SNR range of 5–30 dB. The results clearly demonstrate that the NIRNet algorithm outperforms traditional channel estimation techniques based on SD, SCAMPI, LDAMP, and FCDAMP in terms of NMSE. This superior performance can be attributed to the LSM used in NIRNet, which retains more structural features of the channel matrix compared to conventional Bernoulli random matrices. The LSM’s ability to preserve these structural characteristics enables NIRNet to more effectively capture the underlying channel dynamics, leading to more accurate reconstruction of the channel matrix. This distinctive advantage of NIRNet significantly enhances its performance, particularly in high-noise environments, where maintaining structural integrity of the channel matrix is crucial for accurate estimation.

**Figure 6:**
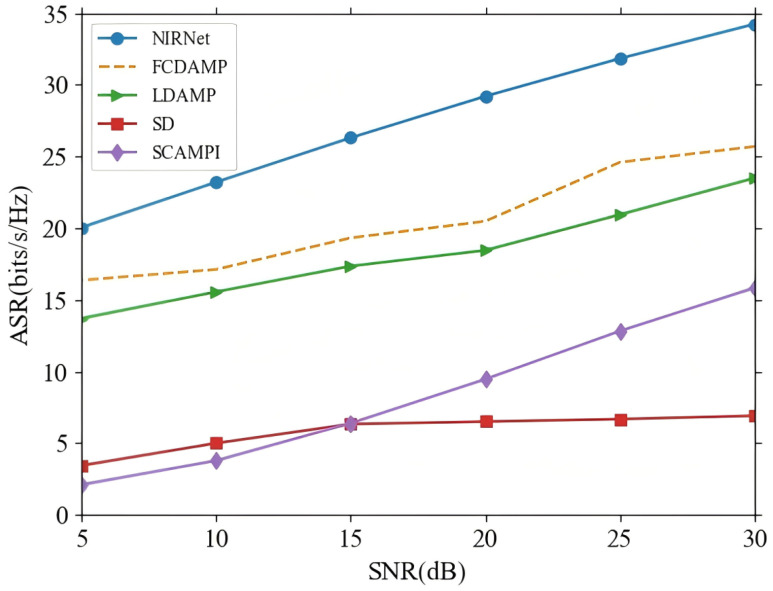
ASR performance comparison between NIRNet and alternative methods.

In addition to NMSE, ASR performance is a crucial metric for evaluating the effectiveness of beamspace mmWave massive MIMO systems. Figure 6 illustrates the ASR performance of the NIRNet algorithm in comparison to several alternative methods, including FCDAMP, LDAMP, SD, and SCAMPI. The results clearly demonstrate that NIRNet achieves a significant improvement in ASR performance over the other algorithms. This improvement is primarily due to NIRNet’s ability to estimate the *k*-th user channel with lower NMSE, which directly leads to more accurate precoding. A more precise precoding matrix results in a substantial enhancement of the overall system performance, particularly in terms of ASR across all *k* users. Therefore, the superior NMSE performance of NIRNet not only contributes to more accurate individual channel estimates but also enhances the overall spectral efficiency of the massive MIMO system.

For practical computing needs, we compare the computational complexity of NIRNet against LDAMP, FCDAMP and LampResNet (as shown in Table 2). The number of multiplication operations required to estimate $H_{k}$ is considered a complexity metric. Multiplication operations in neural networks take place during the convolution operation of the convolution layer. Therefore, for a single convolution layer of a conventional convolution, the computational complexity is expressed as $O\left(B^{2}C^{2}Q_{\mathrm{in}}Q_{\mathrm{out}}\right)$ [26], where *B* and *C* denote, respectively, the spatial lengths of the convolution kernel and output feature map, with $Q_{\mathrm{in}}$ and $Q_{\mathrm{out}}$ representing the number of input and output channels, respectively. For a single octave convolution layer, the computational complexity is expressed as $o\left(\frac{7}{16}B^{2}C^{2}Q_{\mathrm{in}}Q_{\mathrm{out}}+\frac{1}{4}B_{T}^{2}C^{2}Q_{\mathrm{out}}^{2}\right)$, where $B_{T}$ denotes the spatial length of the convolution kernel of the transposed convolution.

## 5. Conclusions

This paper presents a novel NIRNet-assisted channel estimation algorithm for mmWave massive MIMO systems with significant implications for integrated sensing and communications. First, we develop an LSM employing Gaussian-distributed elements that effectively preserves crucial structural features of the channel matrix, establishing a solid foundation for accurate reconstruction, which is potentially beneficial for sensing tasks in future studies. Second, by designing a convolutional layer-based NIRNet that eliminates high-complexity iterative operations, the computational overhead is significantly reduced, offering promise for real-time processing in future sensing applications. Finally, we incorporated a specialized denoising network to refine the initially reconstructed channel, enhancing accuracy particularly under low SNR conditions, which could improve sensing performance in subsequent research. Extensive simulation results confirm our proposed algorithm outperforms existing methods in terms of both NMSE and ASR, while maintaining the better performance with reduced RF chains, substantially lowering hardware costs and power consumption.

## Figures and Tables

**Table 1:** Simulation parameters.

Parameter	Value
Number of antennas at the BS	64
Number of antennas at the user	1
Number of paths	3
Azimuth and elevation	$\phi^{\left(i\right)},\theta^{\left(i\right)}∼U\left[-\frac{\pi }{2},\frac{\pi }{2}\right]$
Path gain	$\alpha^{\left(i\right)}∼\mathrm{CN}\left(0,1\right)$
Training rate	0.001
Number of training samples	16,000
Number of testing samples	8000
Mini-Batch Size	128

**Table 2:** Complexity comparison.

Algorithm	Multiplication Operations
LDAMP	$3.79\times 10^{9}$
FCDAMP	$1.58\times 10^{10}$
LampResNet	$1.48\times 10^{9}$
NIRNet	$9.61\times 10^{8}$

## Data Availability

The data presented in this study are available on request from the corresponding author.
